# Microfluidic assisted synthesis of PLGA drug delivery systems

**DOI:** 10.1039/c8ra08972h

**Published:** 2019-01-15

**Authors:** Sima Rezvantalab, Mostafa Keshavarz Moraveji

**Affiliations:** Department of Chemical Engineering, Amirkabir University of Technology (Tehran Polytechnic) 424 Hafez Avenue Tehran 1591634311 Iran moraveji@aut.ac.ir

## Abstract

Poly(lactic-*co*-glycolic acid) (PLGA) is a biocompatible and biodegradable polymer that recently attracted attention for use as part of drug delivery systems (DDS). In this context, there is an emerging need for a rapid, reliable and reproducible method of synthesis. Here, microfluidic systems provide great opportunities for synthesizing carriers in a tightly controlled manner and with low consumption of materials, energy and time. These miniature devices have been the focus of recent research since they can address the challenges inherent to the bulk system, *e.g.* low drug loading efficiency and encapsulation, broad size distribution and burst initial release. In this article, we provide an overview of current microfluidic systems used in drug delivery production, with a special focus on PLGA-based DDS. In this context, we highlight the advantages associated with the use of microchip systems in the fabrication of nanoparticles (NPs) and microparticles (MPs), *e.g.* in achieving complex morphologies. Furthermore, we discuss the challenges for selecting proper microfluidics for targeted DDS production in a translational setting and introduce strategies that are used to overcome microfluidics shortcomings, like low throughput for production.

## Abstract 2

Poly(lactic-*co*-glycolic acid) (PLGA) is a biocompatible and biodegradable polymer that recently attracted attention for use as part of drug delivery systems (DDS).

## Graphical abstract

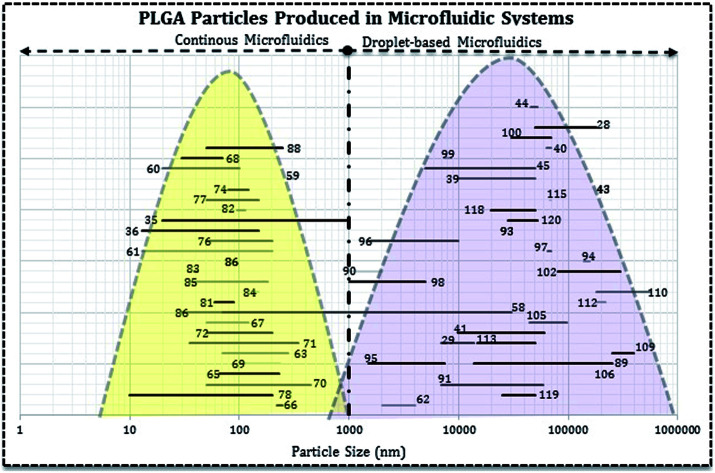

## 1. Introduction

Drug delivery systems (DDS) aim to administer an optimum dosage of drug to the body in order to treat the disease or provide relief pain.^1^ DDS are specially designed to deliver drugs to desired sites in a sustainable manner.^2^ Recently, growing research has been started to find systems that have the features as well as the facility and reproducibility for fabrication methods.^3^ Drug delivery systems include micro- and nano-materials that have applications in tissue engineering, therapy, and even in diagnosis and imaging. For this reason therapeutics with modified activities and features have been the focal points of this extensive research. Polymeric carriers with the capability to tailor and engineer structures are among the most appealing materials for researchers.^4^ Delivery of hydrophilic and hydrophobic drugs is achievable *via* designed polymeric DDS. Problems related to solubility, degradation, and toxicity of various polymers have led researchers towards biocompatible and biodegradable polymers such as aliphatic polyesters.^4,5^

**Fig. 1:**
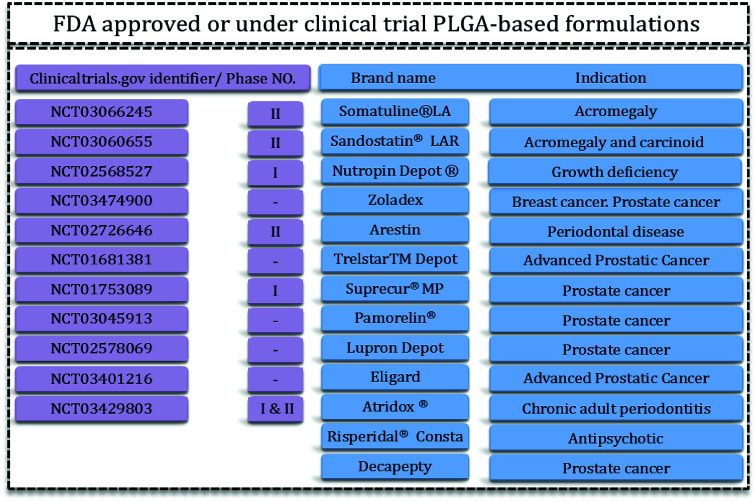
PLGA-based FDA-approved formulations^15,16^ and clinical trials.

PLGA is one of the best characterized biodegradable and biocompatible copolymers that decomposes to nontoxic products (H_2_O and CO_2_) that are eliminated from the body through the Krebs cycle. Typically, PLGA is produced by a catalyzed ring-opening copolymerization of lactic acid (LA) and glycolic acid (GA). Poly(glycolic acid) (PGA) is a crystalline, hydrophilic polymer with low water solubility and fast degradation rate under physiological conditions. On the contrary, poly(lactic acid) (PLA) is a stiff, hydrophobic polymer with low mechanical strength. As a copolymer, PLGA inherits the intrinsic properties of its constitutional monomers. PLGA properties can be tailored for specific applications just by varying the ratio between LA and GA monomers. Due to this phenomenon, PLGA represents a good material for drug delivery systems of many therapeutic agents (*e.g.* chemotherapeutics, as well as antiseptic, anti-inflammatory and antioxidant drugs and proteins). Some of these PLGA based DDS have been approved by the US Food and Drug Administration (FDA) or are in clinical phase trials (Fig. 1).

Drug-loaded PLGA microparticles (MPs)^6–8^ and nanoparticles (NPs)^9–12^ can be synthesized *via* various bulk methods such as salting out, membrane emulsification, single/double-emulsion, nanoprecipitation. Particles produced by conventional bulk methods usually suffer from high batch-to-batch variation and polydispersity. These drawbacks arise due to uncontrollable synthesis method.^13^ For example, all stages of NPs formation including nucleation, growth, and agglomeration take place simultaneously which leads to polydisperse particle formation.^14^

Production process needs good control over surface charge, size and size distribution since these parameters control the drug release rate. In microfluidics the mixing rate, heat, and mass transfer are more precise, synthesis in these miniature devices is more controlled. Moreover, shorter mixing and process time provide another advantage of these small devices; as lower material consumption. These aspects of microfluidics are very advantageous comparing with conventional drug production methods. Therefore these miniature devices can be used to address the conventional methods issues.

The aim of this review article is to collectively encompass the PLGA DDS produced in microfluidics, to address the impact of solvent and microfluidic system in the size and properties of PLGA-based drug delivery systems and pave the way for researchers to choose better system to achieve their goal in the production of drug delivery systems with sophisticated and precise properties.

## 2. Microfluidics

Microfluidics have been defined as devices in which small volumes (micro- or nanoliter) of liquids are processed or manipulated in microchannels to achieve better control on mixing, heat and/or mass transport.^17^ The microchannels are made of various materials such as polymers (*e.g.* polydimethylsiloxane (PDMS) or polyimide), metal (aluminum) and glass capillaries. As microfluidics synthesis allows to tightly control the properties of particles, the technology offers a broad range of advantages over the conventional bulk methods (Table 1).^18–21^ Microfluidics methods can be divided into two main categories based on the flow configuration in the microchannels: droplet-based (segmented) and continues microfluidics.

**Table 1:** The advantages and disadvantages of microfluidics for synthesis^3,13,22,23^

Advantages	Disadvantages
• Tunable particle size	• PDMS is susceptible to solution
• Narrow size distribution	• PDMS is not resistant to high temperature & pressure
• Reproducibility	• Difficult to online purification
• The long duration of continues process	• Difficult online characterization
• More colloidal stability of the produced emulsion	• Microchannel clogging and fouling
• Well-controlled heat and mass transfer	• Limited production scale
• Large surface-to-volume ratio	• The high cost of glass, polytetrafluoroethylene
• Low sample and solution consumption	• Limited scale of aluminum microchannels

### 2.1. Droplet-based microfluidics

Droplet-based microfluidics are used to synthesize microdroplets, emulsions, and microparticles as well as microreactors for nanomaterial production. In the current section, we will provide a brief description of the rules of thumb and principles of droplet generation.

Droplets form because of the instability in the inner flow which breaks into drops. Many parameters are important in the droplet formation, but crucial ones are channel geometry, flow rate, fluid viscosity and surfactant addition. For example, channel design (*e.g.* contraction or the method fluid flows come into contact) and channel diameter are determinant in the droplet formation phenomena and properties. Furthermore, fluid properties such as viscosities and the presence of surfactants are effective parameters in the viscous shear forces, which break the inner stream into droplets.

**Fig. 2:**
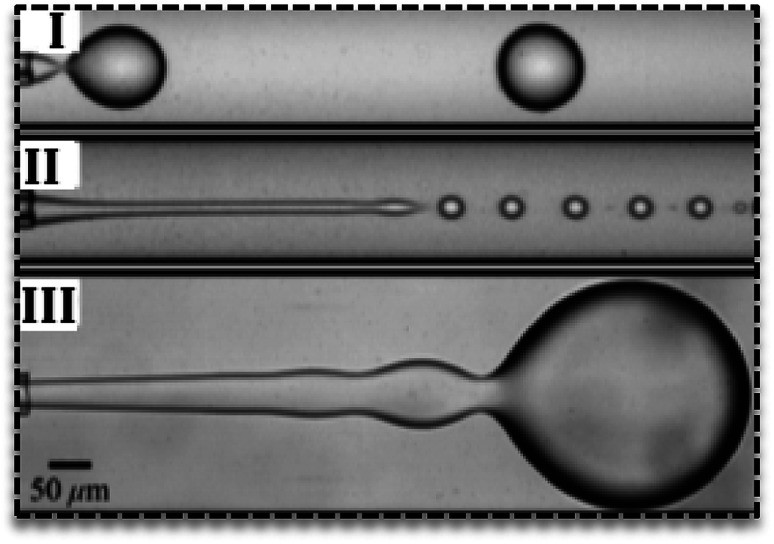
Various flow regimes in the droplet-based microfluidics. (I) Dripping regime, (II) jetting regime, (III) squeezing regime in droplet generation.^25^

Droplets can form in various regimes of flow.^24^ Three main regimes are dripping, jetting and squeezing. The dripping mode has been observed in low flow rates and by an increase in the flow rate, it changes to jetting mode.^25^ Dripping regime produces droplets with narrow size distribution (Fig. 2I) while jetting mode produces polydisperse droplets.^26^ In the jetting regime, droplets are small with higher surface-to-volume ratio^27^ and form far from channels' exit (Fig. 2II).^25^ In the squeezing mode (Fig. 2III), droplets start to grow and plug the continuous phase and consequently by the increase in the pressure of continuous phase they breaks off. Therefore, the squeezing mode is characterized by a fluctuation in the pressure of fluids.

**Fig. 3:**
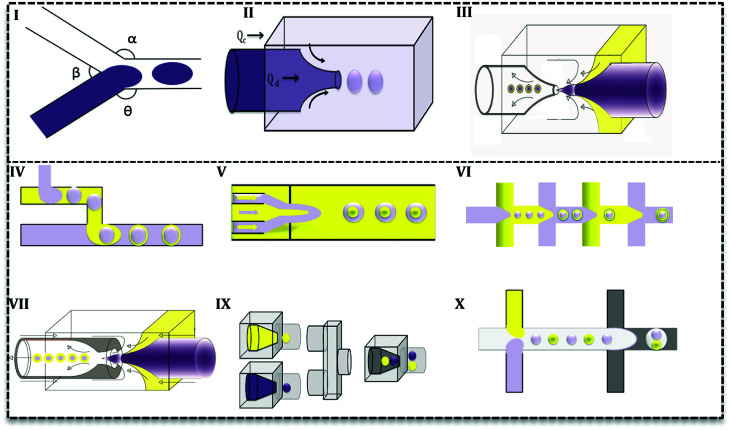
Droplet-based microfluidic designs used to produce emulsions. (I) Cross-flow, (II) co-flow, (III) flow-focusing, (IV) two T-junctions in a row, (V) three concentric channels with one focusing point, (VI) flow-focusing in rows, (VII) combination of co-flow and flow-focusing geometries, (IX) three co-flow, (X) combination of two T-junction and flow-focusing.

Droplet generation as well as mixing in microfluidics can be performed by active and passive methods.^24^ In the active method, an external force (*e.g.* magnetic, electric and *etc.*) is applied to facilitate the droplet formation. In contrast, in the passive mode, two or more immiscible fluids come to contact in a junction and droplets form depending on the properties of the fluid (flow rate ratios, flow conditions, and the geometry of the device).^25^ Various geometries have been evaluated to promote droplet generation with single or multiple cores.^27^ Based on the flow contact, geometries are classified into three main categories: co-flow, cross-flow, and flow-focusing (Fig. 3I–III).^28^

In cross-flow; continuous and dispersed phases meet in a junction with various angles (Fig. 3I) and both stream properties affect the flow and droplets form. Based on the angle of contact, there are various T-junctions (*β* = 180°, *α* = *Θ* = 90°), (*Θ* = 180°, *α* > 90°, *β* < 90°) and (*Θ* = 180°, *α* = *β* = 90°), Y-junction (*β* < 90°, 90° < *α*, *Θ* < 180°). Additionally, designs with more than two inlets have been used as double T-, V- and K-junction.^25^ Out of all junctions, the T- junction is most frequently used since it produces droplets with narrow size distribution.^25,27^ In a co-flow geometry, dispersed phase flows from an inner channel coaxially with the continuous phase in the outer channel with higher flow rate (Fig. 3II). Finally, in the flow-focusing method (Fig. 3III) two phases (dispersed and continuous) flow coaxially and pass through a region with contraction. Regarding the interfacial properties of the phases, at this point, the phase with lower flow rate breaks up into droplets and emulsion forms. Moreover, considering the number of flowing phases, it can produce single or double emulsions.

Aforementioned designs produce single emulsions. Double emulsion can be produced with a combination of them, such as T-junctions in cascade in glass capillary (Fig. 3IV), three co-axial channels with one contraction region (Fig. 3V), multiple flow-focusing designs in series (Fig. 3VI), and combination of a T-junction and a flow-focus (Fig. 3VII). Many researchers have tried to produce multi emulsions in one step (Fig. 3VII),^26,29^ but it is difficult to control flow and the configuration becomes very complex. As it is seen in Fig. 3, microfluidics makes it possible and feasible to generate droplets with varying core size (Fig. 3IX and X) and one core with multiple shells (Fig. 3IX and X). Such droplet morphologies are the basis of microcapsules, core–shell MPs, polymersomes, lipid vesicles, *etc.*^2^

### 2.2. Continuous phase flow microfluidics

In this type, two or more fluids flow side-by-side in microchannels without segmentation or breakup. Researchers try to adopt continuous phase flow for material synthesis due to its reduced mixing time.^30^ A reduced mixing time, resulting from the compression by the outer fluid, is very important in many NPs syntheses since it provides a homogenous condition for NPs formation. As a result, NPs have a more narrow size distribution. Furthermore, uniform concentration, heat, and fluid profile take place in inner fluid and away from channel walls which prevent particle generation close to the channel wall and as a result, it reduces channel clogging.^31^

**Fig. 4:**
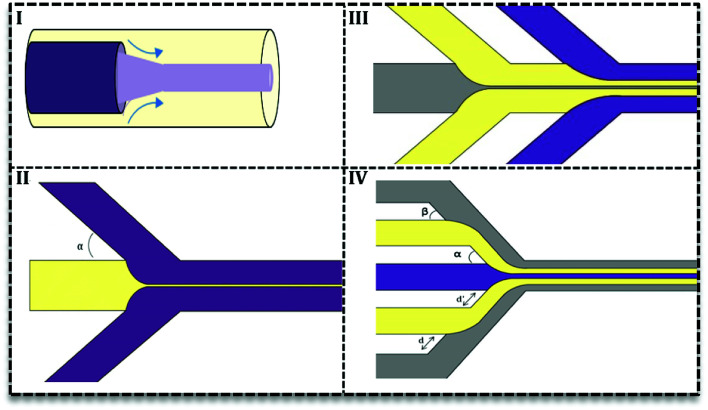
Hydrodynamic Focusing (HF) designs in continuous microfluidics. (I) Co-axial devices, (II) simple HF design in which the central flow is squeezed by two sheath flows from two sides with various angles. (III) HF design with additional sheath-flows to improve flow stability, (IV) multiple HF steps.

There are two types of devices for continuous phase flow microfluidics: the coaxial tube devices which are widely used for inorganic synthesis^32–34^ and the hydrodynamic focusing (HF) devices. The latter are very flexible in design and various subtypes have been recorded (Fig. 4II–IV) based on the number of fluids and angles of contact in the focus point. Fig. 4II shows the simplest HF design with one stream compressed between two streams in various contact angles (*α* ≤ 90°). Multiple HF is possible in a sequential manner (III) or one contraction point (IV). Depending on two angles (*α* & *β*) and distances (*d* & *d*′) this configuration can be used to improve flow stability or avoid central synthesis regime from channels wall.^31^ Recently, 3D designs of HF devices have been investigated in nanomaterial synthesis, in which the inner fluid squeezes between an outer flow horizontally and vertically.^35–37^ Coaxial tube designs (Fig. 4I) were also considered as 3D HF with circular cross section.^27,38^ 3D HF in microchannels with the rectangular cross-section is more complicated and difficult to achieve stable flow. Since in these 3D HF designs particle formation takes place away from channel walls, it prevents clogging and also aggregation of particles.

### 2.3. Microfluidics systems used in PLGA drug delivery systems

In the last two decades, PLGA-based drug delivery systems are being produced in microfluidics. There are several reports of PLGA-based MPs, NPs and microfibers produced using this technology. A wide range of drugs has been loaded into PLGA-based MPs and NPs (*e.g.,* bupivacaine,^39^ risperidone,^40^ ibuprofen,^41^ paclitaxel (PTX),^42^ doxorubicin (DOX),^43^ camptothecin^43^ and *etc.*) in various microchannels such as PDMS microfluidics,^39^ glass capillary,^43^ phenol formaldehyde resin-based microfluidic chip,^44^ aluminum^22^ and silicon.^45^ However, production of microfibers in the microfluidics is not the commonly used synthesis method, especially for drug delivery purposes. The method has been employed to produce microfibers for tissue engineering scaffolds.^46,47^ However, microfibers produced by this method have shortcomings to be used in DDS such as the presence of voids in the microfibers structure^48,49^ and hydrogel nature of the produced fibers due to solidification process.^50^

Considering the microfluidics configuration, various methods of flow and mixing have been employed for NPs production in micro-channels such as segmented or continuous phase flow. Inorganic nanomaterials are synthesized in segmented flow or droplets based microfluidics.^51–57^ In such systems, micro-scale droplets serve as micro-reactors. In the case of PLGA, use of droplet-based microfluidics results into micron size particles. MPs have been synthesized *via* reactions or phenomena that turn emulsions and droplet templates into particles. These phenomena include solvent evaporation or extraction which is not easily achievable in nano-scale. Furthermore, according to the reports, droplet sizes are proportional to the size of the channels. Nano-scale droplets are necessary for NPs production. On the other hand, manipulation and pumping of fluids in such small size channels and capillaries need a large amount of power. Additionally, it is almost impossible to online characterize and control the fluids and droplets properties in nano-scale.

**Fig. 5:**
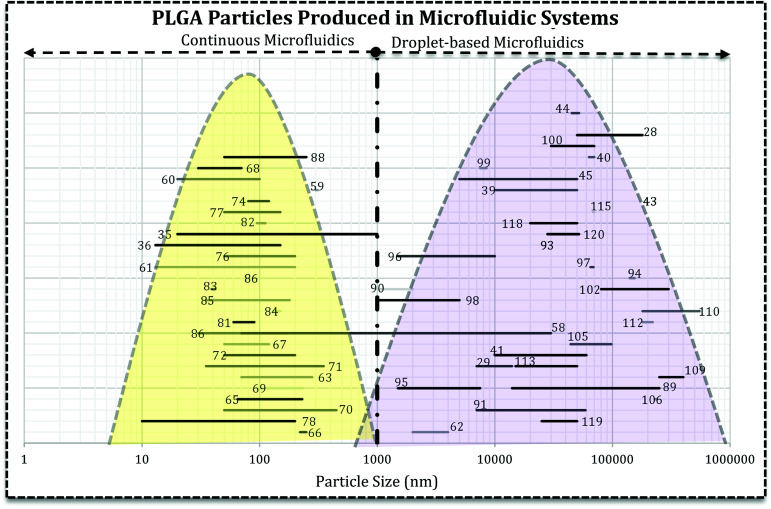
The relationship between microfluidics type and the final size of PLGA-based drug delivery systems. Each line represents the size range of DDS reported in the related reference that is produced in droplet-based or continuous microfluidics. The reader can refer to desired section (*e.g.* Janus MPs Section 4.2) and the references numbers (ref. 89, 116–120) and find the related lines.

Literature survey led us to the idea represented in Fig. 5, *i.e.* the use of droplet-based microfluidics produces PLGA MPs and particle synthesis in the continuous microfluidics will end up at nano-scale. In Fig. 5 each line with a number over it indicates the size range reported by the associated reference article. It can be seen that the type of microfluidics is very important in the scale of the drug delivery system. In more detail, except a few papers, there are not many reports on the PLGA nanoparticles production *via* droplet microfluidics.^26^ For instance, Lee *et al.* succeeded in PLGA micro/nanosphere production through droplet-based microfluidics. They investigated solvent evaporation and extraction effect in the final particles sizes. Fast evaporation of the PLGA droplets in dimethyl carbonate (DMC) produced PLGA MPs (3 to 30 μm).^58^ However, they generated PLGA droplets in dimethyl sulfoxide (DMSO) in silicon oil continuous phase and after infusion with water droplets, solvent extraction led to nano-size (70 to 500 nm) PLGA particle formation. In both methods, particle size increases with the higher concentration of PLGA. In another report, Janus PLGA NPs have been synthesized with T-junction fluidic device consisted of two stainless steel capillaries that enter in a transparent plastic tube. From one inlet PLGA/PLA and Nile red mixture in dimethylformamide (DMF) and the other PLGA and rhodamine 6G in acetone added (with 100 μL h^−1^ flow rate) to an aqueous solution containing 1% PVA (flow rate 10 mL min^−1^). The authors used the same system to load PTX and DOX in PLGA NPs and claimed that the approach overcomes both drugs limitations and offers a high yield of dual-loaded NPs.^59^

Fig. 5 reveals that directly the microfluidic type and indirectly, solvent miscibility, flow rates, and their ratio, the synthesis processes are very crucial in the final characteristics of the drug delivery system and efficiency. It appears that the particle formation process is critical in the final size and size distribution of the carrier. For example, PLGA–PEG solution in a water miscible solvent (acetonitrile, DMSO, DMF) focused with aqueous solution gives rise to self-assembly of polymer chains into polymeric NPs and micelles.^14,60,61^ However, the similar polymeric composition in a water immiscible or semi-miscible solvent (DCM) produces droplets which need a further step to remove the solvent and produce MPs.^62^ For example, change in the solvent from DCM to DMSO and also infusion with water droplets resulted in the sub-micron particles. Although they claimed the droplet-based microfluidic produced nano-scale PLGA particles, the infusion of polymer mixture (water–miscible solvent) with water droplets produced NPs which the precipitation takes place within the droplets.^58^ Additionally, NPs fabricated using DMF as an organic solvent in the T-junction droplet producing a fluidic chip with very low flow ratio.^59^ It can be concluded that precursor's properties and the configuration of streams in microchannels have the decisive influence in the formation process. Although the device resembles droplet-based microfluidics, comparison of dispersed and continues flow rates brings dropwise nanoprecipitation (in the bulk method) into mind. Moreover, for NPs production solvent can significantly affect the properties of drug loaded systems (size and size distribution).^63,64^ Solvents with higher diffusion coefficient produce NPs with smaller size and narrower size distribution.^64^

PLGA NPs produced in continuous microfluidic are in submicron size with low encapsulation efficiency and drug loading. This challenge is associated with water miscibility of the solvents. During the NPs formation, as a result of solvent displacement, polymer chain come together and form smaller particles. However, a huge part of agents is lost at the same time during solvent displacement. Recently, in an interesting approach, Xu *et al.* used glass capillary droplet-based microfluidics in which emulsion was generated by the combination of two solvents; DMSO and DCM.^65^ Although the organic phase forms droplets, one of the solvents (DMSO) displaces into aqueous phase while DCM entraps the drug (DOX) within the droplets during NPs formation, avoiding drug lost. In agreement with the statement, encapsulation efficiency (48.5, 49.9, and 56.9%) and drug loading (9.7, 10.0, and 11.4%) increase in NPs with increase in the DCM ratio in solvent mixture (*V*_DCM_/*V*_DMSO_: 0, 0.05, 0.1).

Taken together, the particle formation process is the basic determining factor of the DDS size. In the continuous microfluidics, PLGA particles form by a nanoprecipitation process in the interface of water–miscible organic solution (middle stream) with the aqueous stream from both side and even sometimes from up and downside. This process is very fast and happens in nano-scale and eventually NPs form. However, in the case of droplet microfluidics, PLGA polymer with drug or agent dissolved in a water–immiscible (or partially miscible) solvent produce template emulsion or droplets that typically evaporated to remove the solvent and produce MPs. As a result, the organic phase solvent and microfluidics configuration regulate the particle formation process, pace and consequently the produced particle size.

## 3. PLGA-based nanoparticles

As it has been mentioned in the previous section, PLGA NPs synthesis in continuous flow microfluidics is accomplished by the nanoprecipitation process between two phases that flow alongside. In this process, a material solution containing polymer and drugs in a water–miscible solvent (*e.g.* acetone, acetonitrile, ethanol, or methanol) is compressed within a non-solvent phase such as an aqueous solution containing surfactants.^66^ The solvent is miscible in non-solvent and transfers between two phases which leads to NPs formation.^30^ Various kind of microfluidics (2D HF to 3D, laminar flow to turbulent jet) has been used to produce PLGA-based NPs.

**Fig. 6:**
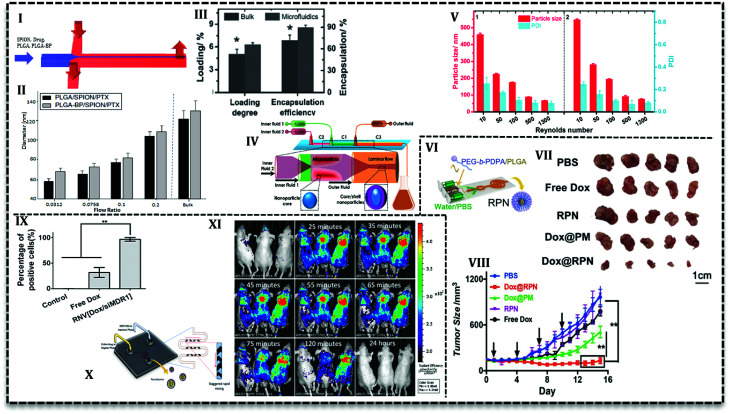
PLGA-based NPs produced in continuous microfluidics. (I) Schematic of 2D HF microfluidics used to load SPION and PTX in BP-PLGA NPs. (II) Diagram of NPs size as a function of flow ratio in the microchannels. NPs size grows with the rise in flow ratios. (III) Drug loading and encapsulation efficiency are higher for NPs prepared by microfluidics compared with the bulk method. (IV) Schematic of two sequential glass capillaries used for the preparation of core–shell NPs with homogeneous size distribution through rapid mixing. (V) NPs size and polydispersity index drop significantly in higher Reynolds numbers. (VI) The polymeric solution consisted of drug and PEG-*b*-PDPA/PLGA in DMF and trifluoroethanol (TFE) is focused from two sides with an aqueous solution containing PBS in 2D HF microfluidics. (VII) Images of *ex vivo* tumors treated with PBS, free DOX, and rigid pH sensitive NPs (RPN), DOX-loaded micelles without PLGA core (DOX@PM), and DOX-loaded RPN (DOX@RPN). (VIII) Tumor volume treated PBS, free DOX, and RPN, DOX@PM, and DOX@RPN indicate that core/shell NPs with PLGA core have higher antitumor activities among other groups. (IX) Co-incubation of free DOX and siMDR1 and Dox-loaded NPs with MCF-7/ADR cells for 2 h show that NPs are completely (100%) taken up while free DOX cellular uptake is 30%. (X) Micromixer with a herringbone pattern to improve mixing efficiency used for curcumin-loaded PLGA–lipid NPs. (XI) *In vivo* evaluation with C4-2B xenograft mice shows that after 24 hours is removed completely from the body.

The simple yet convenient 2D HF type allows fabrication of multi-drug loaded PLGA NPs with desired properties. For example, bisphosphonate conjugated PLGA (BP-PLGA) NPs loaded with superparamagnetic iron oxide nanoparticles (SPIONs) and PTX are produced in a PDMS based chip (Fig. 6I) to be used in chemotherapy, hyperthermia, and MRI diagnosis.^67^ Results show that NPs produced in microfluidics (in the range of 40 to 100 nm) is smaller in comparison with bulk method (≥120 nm). Furthermore, it is possible to control the properties of NPs with flow conditions in 2D HF microfluidics. For example, by an increase in the flow rate ratio (the ratio of the organic phase containing NPs precursors to aqueous phase), final NPs size increases (Fig. 6II). Additionally, this ratio can affect drug release profile, as in lower ratios, NPs are smaller and more compact, consequently, drug release is slower. Results from *in vivo* analysis with a bone metastasis model (MDA-MB-231) mice showed that tumor growth suppression and apoptosis level enhanced with targeted microfluidic NPs. In a recent study,^68^ it was shown that an increase in the flow ratio from 0.025 to 0.125 means size of non-targeted curcumin-loaded PLGA NPs increase from about 30 nm up to 70 nm. The authors stated that the higher the ratio, the broader the size distribution of produced NPs. Results from *in vitro* analysis exhibited that compared with free curcumin, microfluidically produced NPs enhance the antitumor activity of the drug toward leukemia Jurkat cells. As it's seen, flow ratio is an effective parameter in the synthesis process and lower flow ratio produces smaller NPs with narrow size distribution. However, lower flow ratio means the slower flow of NPs precursors and an undesirable consequence of slow flow is low efficiency which is the intrinsic characteristic of low flow rates.

Another effective parameter is mixing time and determined by the design of the microfluidic chip. Mixing time controls the particle formation and also final properties and the amount of NPs. In 2D HF microfluidics with microchannels, flows are laminar; mixing is based on diffusion which takes place in the interface of phases. In order to investigate the effect of design on mixing time and also NPs properties, PLGA–DOX solution in the mixture of DMF and trifluoroethanol (TFE) was introduced into three PDMS based chips: 2D flat HF, 3D arc, and 3D double spiral.^69^ Simulation results represented that the mixing time as a function of flow rate can be prolonged by an increase in flow rate. Moreover, 3D designs have a shorter mixing time due to the shortened mixing distance. For instance, in the same flow rate (2.5 mL h^−1^) mixing time decrease for 2D flat HF, 3D arc and 3D double spiral (29, 16, and 14.5 ms, respectively). However, encapsulation efficiency (*ca.* 50%) and cellular uptake with MCF-7 and HeLa cells are reported only for 100 nm NPs produced in origami chip. DOX-loaded PLGA NPs were more taken up with cancer cells and showed higher cytotoxicity compared with free DOX. In another conducted research, Liu *et al.* used 3D coaxial flow in the capillary glass to minimize the mixing time. They increased the flow rates which caused flow regime transfer from laminar to turbulent jet.^63^ An additional distinguishing feature of their design is that organic phase (PLGA solution and PTX) flows as outer fluid near the wall of the channel and aqueous phase flows in the central glass capillary. Higher drug loading and encapsulation efficiency (Fig. 6III) reported for microfluidic NPs compared with the bulk method. The results attributed to the fact that volume ratio between polymer precursor and an aqueous solution for the microfluidics system is fixed and higher than bulk synthesis (the ratio is low and increases gradually during the process). PLGA NPs with the size ranges of 100–210 nm can be produced with mass production rates up to 242.8 g per day. In a similar configuration with one more capillary glass (Fig. 6IV) PTX and sorafenib (SFN) (anti-angiogenic drug) are assessed to be loaded in core–shell NPs.^70^ Results declare that drug loading increases for PTX (from 6.7% to 42.6%) and SFN (from 6.2% to 45.2%) with a sequential configuration in comparison with single step process. As it's seen in Fig. 6V, the higher the flow rates (higher Reynolds number) the smaller the NPs size.

Microchannel size is another effective parameter in the formation and properties of NPs. In larger diameter channels the ratio drops and NPs size increases. The smaller size of the NPs produced in microfluidics is a result of a higher surface-to-volume ratio offered by these devices. In a 3D microfluidics fabricated out of commercially stainless steel capillary with three inlets, dexamethasone and ribavirin encapsulated in NPs with size range 35–350 and 50–200, respectively.^71,72^ According to the outcomes, by a decrease in the internal diameter of the channel from 600 to 130 μm, NPs size dropped from about 133 to 28 nm. Researchers mentioned that easily assembled device has the capability to be used in a series of parallel designs for mass production up to 2.4 kg NPs per day.^71^

### 3.1. PEG–PLGA NPs

PEGylation is the process of polyethylene glycol (PEG) chains conjugation on the molecules or microstructures. The NPs consists of PLGA core and PEG chains on the surface offer a wide range of advantages; *e.g.* long circulation time due to immune system evade, small size around 20–100 nm, higher solubility, and stability, capability for drug encapsulation, good degradability, and biocompatibility.^73^ PEG chain conjugation to PLGA has been investigated for a long time. PLGA–PEG copolymers assemble into NPs or micelles in the aqueous phase. Therefore, the precipitation in the aqueous phase to produce NPs is being used for a long time in the bulk method and it has been adapted to the microfluidic system in the last decade.

For the first time, Karnik *et al.* reported nanoprecipitation of PLGA_15k_–PEG_4k_ in 2D HF microfluidics.^14^ They reported that NPs size and drug encapsulation efficiency are affected by flow rates and composition of phases, *i.e.* incorporation of PLGA increases encapsulation efficiency from 28% to 51%. Incorporation of PLGA or PLA increases the NPs size in bulk method. However, to prevent the size enlargement, PLA modified with prodrug (platinum(iv) [Pt(iv)]) has been incorporated to PLGA–PEG for drug release control.^74^ The polymer solution used to load hydrophilic cisplatin and hydrophobic docetaxel into NPs in a 2D HF microfluidic. Nanoprecipitation with identical condition produced smaller NPs in a microfluidics (∼100 nm) compared with the bulk method (greater than 150 nm). In a similar study, cisplatin prodrug (as conjugated to PLA backbone) and free irinotecan loaded into PLGA–PEG NPs to target prostate-specific membrane antigen (PSMA) overexpressing prostate cancer cell using *S*,*S*-2-(3-[5-amino-1-carboxypentyl]-ureido)-pentanedioic acid ligand.^60^ Addition of cisplatin–PLA to the solution of PLGA–PEG with irinotecan increased its encapsulation efficiency from 10% up to 44%. For both studies, results from *in vitro* analysis with LNCaP cells showed that the combination of drugs increases the cytotoxicity toward the cells compared with single drug-loaded NPs. In the following study, the authors used two-stage microfluidics for mixing and production of doxorubicin-loaded PLGA–PEG targeted NPs in a fully integrated microfluidic device.^61^ They could produce a library of NPs with various surface properties, ligand densities, size, and molecular weight to evaluate *in vitro* and *in vivo.* Addition of 14% mole of targeted polymer to PLGA–PEG mixture increases the LNCaP cells uptake and tumor accumulate up to 3.5-fold compared with bare PLGA–PEG NPs. In order to promote the cellular uptake, pH-sensitive NPs have been produced in a similar 2D HF microfluidics (Fig. 6VI) which can escape endo/lysosomes and overcome drug resistance.^75^ The core–shell NPs consisted of DOX-loaded PLGA core and poly(ethylene glycol)–poly(2-(diisopropylamino)ethyl methacrylate) (PEG-*b*-PDPA) diblock copolymer shell. The authors claimed that upon the translocation in the acidic endocytic, the residual parts of shell produce positive charges over the PLGA core and help lysosomal escape. Results for *ex vivo* and *in vivo* analysis with MCF-7/ADR tumor-bearing mice reveal that in comparison with free DOX, the NPs significantly suppress the drug-resistant tumor growth (Fig. 6VII and VIII).

To produce as small as possible NPs with homogeneous size, PLGA–PEG self-assembly investigated in 3D hydrodynamic focusing microfluidics with sequential inlets.^35,36^ Results disclosed that not only the concentration of polymer is important but also polymer molecular weight is a contributing factor in the final size of NPs. For instance, with an increase in PLGA molecular weight (10 to 90 kDa) the produced NPs size increases (∼26 to 150 nm). Moreover, in the same molecular weight (10 kDa) with increase in the concentration (10 to 50 mg mL^−1^) the NPs size increases (13 to 26 nm, respectively). The 3D microfluidics has reduced the fouling and clogging since reaction takes place far away channels wall.^35^ However, the 3D microfluidics is complicated and it is difficult to achieve stable flow and reproducible manner. To increase the throughput, 8 parallel 3D devices in one chip, they reduced the batch time (for 25 mg) from 5 h to less than 20 minutes.^36^

Low throughput of PEGylated PLGA NPs produced by these miniature devices is an important challenge and it has been tried to overcome in various research. In one attempt a 3 layer PDMS microfluidics with 100 channels fabricated that produced methoxyl PEG–PLGA (MPEG–PLGA) up to 0.5–2.0 mL h^−1^ polymer flow rate with narrow size distribution.^76^ Another 3D HF microfluidics fabricated with parallel polyimide films which can tolerate up to 16 MPa with high throughput up to 331 g per day of PEG–PLGA NPs.^77^ Lim *et al.*^78^ designed a turbulent jet micromixer with a higher flow rate and consequently high production more than 3 kg per day which is the highest throughput achieved up until now.^3^

### 3.2. Lipid–PLGA NPs

Lipids are hydrophobic or amphiphilic molecules which also can be used to modify molecules. They have attracted attention for PLGA surface modification.^79^ These core/shell structures have hydrophobic cores and hydrophilic tails of lipids. These structures are capable of hydrophobic drug loading and have prolonged circulation time compared to PLGA NPs.^79,80^

Recently, lipid–PLGA NPs have been produced in the microfluidic device to control the reaction and properties of the particles. In agreement with results, lipid–PLGA NPs with a smaller size (∼62.5 and ∼87 nm) are produced in lower total flow rate (41, 246 mL h^−1^, respectively). Moreover, cellular uptake evaluation with A375 cells (human melanoma cell line) indicated that smaller lipid–PLGA NPs are internalized more efficiently compared with larger counterparts.^81^ Zhang *et al.* synthesized dual drug (DOX and combretastatin A4 (CA4)) loaded PLGA NPs with mono- and bi-layer lipid shells in a two-stage HF microfluidics.^82^ Cellular uptake analysis with HeLa (cervical cancer cells) and HUVEC cells (Human umbilical vein endothelial cells) showed that NPs with monolayer lipid was taken up more than bilayer counterparts and even free drugs. Furthermore, similar findings observed with *in vivo* and *ex vivo* analysis, *i.e.* monolayer NPs exhibited improved anticancer activity and faster tumor accumulation. The same procedure adapted to produce lipid–PLGA and lipid–water–PLGA NPs loaded with doxorubicin in PLGA core and CA4 in the shell, respectively. Rigidity analysis showed that NPs with a layer of water between polymer and lipid layers are more flexible than the bi-layer counterparts. *In vitro* analysis (with HeLa and HUVEC cells) verified the results from a molecular dynamics simulation that revealed rigid NPs have enhanced cellular uptake compared with free drugs and flexible NPs.^83^ According to the findings that rigid lipid–PLGA NPs exhibit higher cellular uptake, similar morphology adapted to load hydrophilic agent (siRNA) in water core, hydrophobic drug (DOX) in PLGA layer and lipid shell. The core/shell morphology enabled co-delivery of siMDR1 (the siRNA sequence against the multi-drug resistant protein) and doxorubicin and performed an enhanced gene knockdown efficiency compared with lipofectamine^2000^. *In vitro* analysis (Fig. 6IX) by MCF-7/ADR cells showed that cellular uptake of the lipid–PLGA NPs (∼100%) is much higher than free DOX (30%). The NPs was surprisingly effective in tumor growth inhibition for mice treatment in comparison with and free drug, free gene.^84^

Lipid shells not only used to load drugs also improved the stability of polymeric NPs but also used to enhance quantum dot (QD) nanocrystals hydrophilicity and biocompatibility in biological environments. In a conducted study, PLGA solution in acetonitrile focused in a 2D HF microfluidics with lipid solution (aqueous solution of lecithin and 1,2-distearoyl-*sn-glycero*-3-phosphoethanolamine-*N*-[carboxy(polyethylene glycol)] (DSPE–PEG)) and afterward mixed in a Tesla microstructure to produce lipid–PLGA NPs. Additionally, in order to load quantum dots for diagnosis application, they used the same arrangement and solutions in which the aqueous phase contained lipophilic quantum dots (dissolved in tetrahydrofuran).^85^ Results exhibited that rapid mixing in Tesla micromixers produces monodisperse NPs (35–180 nm) since it improves mixing efficiency. Results with various experiment revealed that lipid–PLGA NPs with 40 nm size have the most stable form and also lipid : PLGA (*e.g.* 1 : 10 to 1 : 1000) ratio is not effective in the size of NPs. Another imaging agent (gold nanocrystals (AuNCs)) with two therapeutic loaded in lipid–PLGA NPs. With an interesting approach, PLGA which was functionalized with AuNCs forms a hydrophobic core loaded with DOX and a lipid layer contains SRF, lipid shell composed of ordinary phospholipids and PEGylated phospholipids.^86^ Another interesting point about the study is the use of a 3D microfluidic chip with three inlets to increase the production and control the size of NPs. Drug release analysis reveals a sequential release of drugs; SRF release followed by DOX release and *in vivo* evaluations represent higher accumulation in the tumor site. Kim *et al.*^87^ used the same chip to investigate the flow pattern and condition on the size and mass production of lipidic PLGA NPs. In the study, organic solutions (acetonitrile containing polymer) in the middle inlet and aqueous phase (lecithin and DSPE–PEG) in the outer inlets generate micro vortices. NPs sizes are affected by flow rates and Reynolds number, as in higher Reynolds numbers (Re = 150, 75), NPs size decreases (55 nm and 81 nm, respectively). The authors claimed that their microfluidics has higher productivity (up to 3 g h^−1^) which is 1000-fold of the conventional 2D HF microfluidics. Gdowski *et al.*^88^ used a micromixer with herringbone pattern (Fig. 6X) to promote the mixing of PLGA and curcumin in acetonitrile with an aqueous phase containing DSPE–PEG. They optimized NPs size to 102.11 nm with 4.4% drug loading and 58.8% encapsulation efficiency. However, *in vivo* assessment with mice bearing prostate cancer cells (C4-2B) showed that NPs are completely removed from the body after 24 h (Fig. 6XI).

## 4. PLGA-based microparticles

Microparticles have great importance in biomedical applications because of their capability in the delivery of a broad range of drugs, higher encapsulation efficiency, controlled and stimuli release.^25,39,89,90^ Due to the good properties of PLGA for biomedical applications, researchers have considered different geometries, chips, and configurations to produce PLGA-based microcarriers with tunable size and morphology. Table 2 summarizes PLGA MPs production in various microfluidic systems with different agents to be used in drug delivery systems. The current section covers PLGA MPs produced in microfluidic systems.

**Table 2:** PLGA MPs synthesized in microfluidics

Microfluidic	Channel size	Chip geometry	Number of core	MPs size (μm)	PLGA (L/G)	Agent	Ref.
PDMS	100–200	Flow-focus	1	10–50	85 : 15		39
Glass capillary	Inner tube: 30–50 μm middle tube: 100 μm	Co-flow	1–3	∼210	50 : 50	Doxorubicin hydrochloride	43
PDMS		Flow-focus	1	3–6	50 : 50	—	23
PDMS	Height 100 μm width 200 μm	T-junction	1	50–65	75 : 25	ac-Rb1 (6′′-*O*-acetylginsenoside Rb1)	91
PDMS	—	Flow-focus	1	20.7 ± 1.56	50 : 50	H_2_O_2_	92
Aluminum	Height 50 μm width 100 μm	Cross- junction	1	42.8 ± 2.3	—	CdSe/ZnS	22
PDMS		Flow-focus	1	∼28	50 : 50	Tanshinone IIA	93
Capillary	700 μm & 1 mm	Flow-focus	1	≈145	75 : 25	Dexamethasone + latanoprost	94
PDMS	Height 20 μm width 30 μm	Flow-focus	Hollow MPs	<7	50 : 50	Celecoxib (CEL) & sorafenib (SFN)	95
Glass capillary	—	Flow-focus	1	—	50 : 50	Gas	96
Fluoroelastomer SIFEL	100 μm	Flow-focus	1	67.0 ± 1.6	75 : 25	—	97
Glass capillary	50 μm at orifice	Flow-focus	1	1–2	50 : 50	Insulin	90
FF nozzles	100 μm	Flow-focus	1	4–12	50 : 50	Green fluorescent protein	29
Brass	Diameter 6.0 mm length 10.0 mm	Flow-focusing	1	1–5	85 : 15	—	98
FF nozzle	—	Flow-focusing	1	3–6	50 : 50	Lidocaine	99

Typically, MPs in microfluidics are produced *via* template droplets that turn into MPs through various reactions or in the case of PLGA, by solvent evaporation or diffusion.^25^ Rapid processing in microfluidics needs a volatile solvent to evaporate rapidly as well as dissolve organic component. For this aim, organic solvents such as DMC, chloroform, and toluene are frequently used to produce droplets in an aqueous continuous phase. The solvent is important since it can affect the production process or even the properties of the final particles. For instance, encapsulation efficiency, an initial burst release of enoxacin (ENX)-loaded PLGA MPs synthesized in a PDMS microfluidics is controlled by changing the solvent from DMC to dichloromethane (DCM).^100^ MPs produced by PLGA dissolved in DMC exhibited higher encapsulation efficiency (56.5%) and initial burst release (14.8%) compared with DCM (encapsulation efficiency 15.4%, initial burst release: 12%). The concentration of the organic phase is effective in the size of particles. With the increase in PLGA concentration (1, 3, and 5 wt%) in DMC, MPs size increases from 140 to 160 μm.

Stability investigation of PLGA MPs has been carried out by various researchers.^101^ Tu and Lee investigated the effect of PLGA composition and the pH of the inner phase of water-in-oil-in-water (W/O/W) double emulsions. They showed that basic phase in double emulsion PLGA microcapsules enhances the surface activity and consequently improve colloidal stability.^102^ Furthermore, it has been proven that monodisperse MPs produced in microfluidics have better colloidal stability in aqueous dispersion when compared MPs produced a bulk method that tends to aggregate in storage.^23^

Droplets and emulsions stability is also important in the final characteristics of MPs which can be affected by the chip properties and configuration. For instance, PDMS is hydrophobic and it's difficult to produce droplets with varying hydrophobicity. Some modifications have been proposed and used^103,104^ such as immersion in the poly(vinyl alcohol)/glycerol solution to produce PTX loaded poly(l-lactic acid) (PLLA) microspheres.^42^ It is of the utmost importance to consider its characteristics in droplet and emulsion productions. Hydrophilic PDMS changes to hydrophobic in contact with organic solutions such as dichloromethane. To prevent this phenomenon which circumvents droplet and particle formation, Xu *et al.* used T-junction flow-focusing geometry. In the configuration, water is in contact with MPs wall which surrenders the dichloromethane phase. Bupivacaine loaded PLGA MPs in the range of 10–50 μm size produced by capillary device release drug more slowly and also have significantly smaller initial burst release in comparison with conventionally produced particles.^39^

**Fig. 7:**
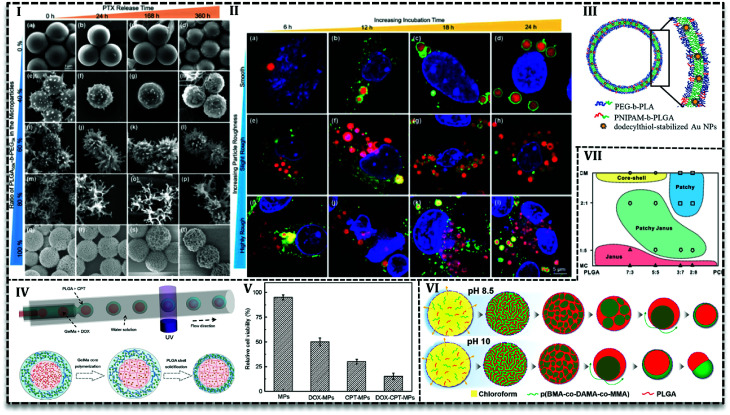
PLGA-based MPs produced in droplet-based microfluidics. (I) SEM images show the drug release of PLGA_50k_-*b*-PEG_5k_/PLGA_100k_ polymer MPs with varying polymer ratio in different times. Authors concluded because of diffusion drug release mechanism MPs surface morphology is stable over time. (II) Cell lysosomes were stained with Lyso Tracker Green DND-26 (green) while blue Hoechst was used to label the nuclei. Confocal Laser Scanning Microscope image demonstrates cellular uptake of Nile red labeled MPs with B16-F10 melanoma cell that indicates the MPs are taken up by the cancer cells and the MPs can act as drug carriers to successfully transport hydrophobic drugs to the cancer cells. (III) Schematic of the photo- and thermoresponsive MPs composed of a bilayer of PEG-*b*-PLA and PNIPAM-*b*-PLGA diblock copolymers, containing dodecylthiol-stabilized gold nanoparticles to induce responsive release. (IV) Schematic of W/O/W double emulsion templates generation in a capillary microfluidics and the illustration of the drug-loaded GelMa–PLGA core–shell MPs formation in the device. (V) *In vitro* analysis of the human colon cancer cell line (HCT116 cells) treated with unloaded MPs, only DOX-loaded MPs, only CPT-loaded MPs, and DOX-CPT-*co*-loaded MPs for 24 h revealed that antitumor effect of dual drug loaded MPs is higher than individual drugs. (VI) Schematic of the effect of in the final morphology of the Nile red and coumarin loaded core–shell pH-responsive MPs formation from a homogeneous chloroform drop containing p(BMA-*co*-DAMA-*co*-MMA) and PLGA in the aqueous phase with pH 8.5 and 10. (VII) Various structures produced by different volume ratio of the solvents (dichloromethane/dimethyl carbonate) and the mass ratio of PLGA/PCL (the total concentration was 40 mg mL^−1^).

Various studies have been conducted to investigate the microfluidics capability in the control of MPs shape and morphology. In this regard, PLGA-based MPs fabricated with various shape such as honeycomb,^105–107^ Golf-ball,^106^ snowman microcapsules,^108^ porous microbeads.^109^ Hussein *et al.* reported that surface texture is easily controllable using polymers with various hydrophobicity in a microfluidics system.^62^ They prepared PLGA_100k_ blend with PLGA_50k_-*b*-PEG_5k_/PLGA_100k_ or PLGA_10k_-*b*-PEG_20k_/PLGA_100k_ and PTX as a solution (10 mg mL^−1^) in DCM as a dispersed phase in the aqueous phase (5 mg mL^−1^ PVA) with varying ratios in a glass capillary. Results indicated that surface texture is controllable with the blend ratio and consequently it affects encapsulation efficiency and releases kinetics. For example, with an increase in the PEG content (0 to more than 60 wt%) particles surface changes from a smooth to bumpy appearance and finally breaks into nanoscale micelles. Moreover, encapsulation efficiency drops with an increase in the PEG content, *e.g.* encapsulation of PTX is reported about 92% for neat PLGA_100k_ (PEG = 0), while in the case of neat PLGA_50k_-*b*-PEG_5k_ it is about 64%. The authors attributed the results to the fact that higher hydrophobic content of polymer promotes more PTX encapsulation. PEG content also enhances the PTX release, as the highest drug release profile (Fig. 7I) is observed for 80% of PLGA_50k_-*b*-PEG_5k_ and the lowest for neat PLGA_100k_. On one side, the more the hydrophilic content, the more the water adsorption and on the other side the more the PEG content, the higher the roughness and consequently, the more interfacial area available for water diffusion. All together promote drug release for particles with a higher amount of PEG in the polymer blend.

From another point of view, microfluidics provides advantages to load agents with various properties in polymeric matrixes. It enables inorganic material loading into PLGA MPs and hybrid MPs production. For instances, the cross-junction microfluidic system has been applied to encapsulate CdSe/ZnS QDs in PLGA MPs with the size of 180 to 550 μm. PLGA along with CdSe/ZnS QDs (4 ± 0.5 nm) in chloroform produces droplets in an aqueous phase containing PVA (1 wt/v%) and chitosan (0.5 wt/v%) in a flow focusing microchip.^110^ W/O/W double emulsion template produced in a capillary microfluidics consisted of an inner aqueous phase containing 10 wt% PEG with 8-hydroxyl-1,3,6-pyrenetrisulfonic acid trisodium salt (a green dye), and sulforhodamine B (a red dye), middle oil phase containing PEG-*b*-PLA with poly(*N*-isopropylacrylamide) (PNIPAM)-*b*-PLGA (as thermosensitive polymer) and dodecylthiol-stabilized gold NPs (a photosensitive agent) in a mixture of chloroform and hexane, outer aqueous solution containing 10 wt% PVA. After evaporation, polymersome produced with a double polymer layer (Fig. 7III) in which gold NPs are entrapped in the PNIPAM-*b*-PLGA part. By examination and optimization with various amounts (2, 5, and 10 wt%) of PNIPAM-*b*-PLGA, they concluded that 5 wt% is needed to produce thermosensitive polymersome. On the other hand, a higher amount of thermosensitive polymer results in defects and finally ruptures of the polymersome. The release mechanism of polymersome induced by temperature and laser illumination indicated that in thermoresponsive release pores form in the polymersome and releases the load gradually. However, laser triggered release starts with local hot spots formation which finally results in the rupture of bilayer.^111^ PLGA microspheres containing TiO_2_ NPs on the surface produced in FF chip. PLGA and titanium tetraisopropoxide (TTIP) in DCM generated droplets in aqueous solution 90 wt% of glycerol and 0.5 wt% of PVA. TiO_2_ forms upon the contact with aqueous phase as a result of TTIP hydrolysis also make MPs with a wrinkled surface. With the increase in the mass ratio of TTIP/PLGA (4/30 to 8/30), surface wrinkles get deeper and after 12/30 particle changes to non-spherical. Tanshinone IIA incorporated into MPs as a model drug with encapsulation efficiency higher than 80% and results from *in vitro* drug release showed the deeper the wrinkles on the surface the higher drug release rate.^93^

### 4.1. Core–shell microparticles

Core–shell MPs or microcapsules contain gas, liquid or solid core (or multiple cores) covered up in the shell.^25,112^ These morphologies offer a broad range of advantages such as dual or multiple drug delivery, controlled and prolonged release, protection against active agents.^113^ Moreover, the core–shell structure can deliver chemically active agents and protect them from body proteins, immune system, and degradation. Microchannel-based synthesis offers unique opportunities to produce core–shell, multiple cores in one shell and even multiple shells on one core. It is done through solidification of single/multiple emulsions.

For instance, Martín-Banderas *et al.* encapsulated gentamycin sulfate (GS) in PLGA MPs with one or more core and microcapsules.^114^ They used an FF configuration with PLGA and drug solution focused with an aqueous solution to produce MPs. They used the same configuration with multiple concentric needles with air in the inner needle to produce microcapsules. They reported high drug loading (10 to 30%) and encapsulation (42 to 85%) efficiencies for the microcapsules compared with MPs. In another report, alginate shell on PLGA MPs synthesized in a flow focusing capillary microfluidic used to control the release of rifampicin in the size range between 15 to 50 μm. The core–shell morphology produced from W/O/W double emulsion templates in which consisted of inner aqueous phase (0.5% sodium alginate and 10% (w/v) PVA), middle oil phase (DCM with 20% PLGA) and outer aqueous phase (10% (w/v) PVA and 4% calcium chloride (CaCl_2_)). Both shell and core sizes affect the drug release and by an increase in the MPs size, initial burst release decreases. Moreover, core–shell structure exhibited higher drug content (∼6.4%) than microspheres (4.26 ± 0.54) and also higher encapsulation efficiency (70.47 ± 1.85%) compared with microspheres (46.78 ± 5.89) with similar size (∼50 μm). In agreement with results from other investigations,^65^ the authors attributed the results to shell layer that prevents the drug from diffusion to solvent in the evaporation stage of the fabrication process.^113,115^

PLGA-based MPs containing liquid cores are thermodynamically unstable and shell rupture takes place during solidification and degradation that causes to burst release of the loaded drug. Various strategies have been used to overcome this shortcoming and improve MPs stability. For instance, gelatin methacrylate (GelMa) used as a crosslinking agent to avoid rupture or fusion of cores.^43^ PLGA-based core–shell structure containing DOX hydrochloride and camptothecin (CPT) fabricated by one-step co-flow capillary microfluidics (Fig. 7IV). Double W/O/W emulsion produced in three-concentric capillary tubes. Inner aqueous phase contained hydrophilic drug (DOX hydrochloride) and GelMa (15% w/v) while the outer aqueous phase contains PVA (2% w/v). Hydrophobic drug (CPT) dissolved in DCM with PLGA polymer as a middle oil solution. Emulsions polymerized by UV light to solidify core to produce stable core and release two or more therapeutics sustainably. They achieved the varying size of MPs and core numbers (one, double and triple cores in one shell) with various orifice size and fluid flow rates. Results illustrated that increase in the shell thickness (22, 40, and 60 μm) leads to higher encapsulation efficiency of CPT (∼46, 57 and 61%) and DOX hydrochloride (85, 89 and 93%) and higher drug content (4.06 ± 0.02, 6.17 ± 0.15 and 6.88 ± 0.24). *In vitro* evaluation with human colon cancer cell line (HCT116) (Fig. 7V) and liver cancer cell line (HepG2) revealed the synergistic antitumor effect of two drugs. Antitumor effect of dual drug loaded MPs (less than 20% HCT116 cells and 10% HepG2 cells survive) is higher than individual drugs either DOX or CPT (50% HCT116 cells and 60% HepG2 cells are killed). In another approach, Montazeri *et al.*^92^ improved the PDMS based double flow focusing chip to produce a partially hydrophilic–hydrophobic microfluidic device. They added a surfactant (Silwet L-77®) to the curing agent and prepolymer of the PDMS in chip preparation stage to improve the wettability of the chip. They investigated contact angle of water droplet by the surface of PDMS that led them to choose 0.5 wt% among the various concentration of surfactant (0, 0.2, 0.5 and 0.8 wt%) to produce PLGA based MPs with average 20 μm. Results reveal that modification enables H_2_O_2_ solution delivery as an oxygen generator into islet transplantation over an extended time up to 30 days.

### 4.2. Janus microparticles

Janus or bifacial MPs have excellent properties such as tunable and controllable asymmetry in shape, composition, the capability to load multiple and even incompatible agents.^89,116^ In addition to these properties, recently Janus MPs production in microfluidics attracted more attention because of their facility and capability in the synthesis and control of physical and chemical features.^116,117^

In order to achieve Janus MPs, two-face droplets are necessary which will solidify to MPs after droplet consolidation. Therefore, two miscible fluids form droplets in a third immiscible fluid. Min *et al.* dissolved PLGA and poly(butyl methacrylate-*co*-(2-dimethylaminoethyl) methacrylate-*co*-methyl methacrylate) (p(BMA-*co*-DAMA-*co*-MMA)) in an organic solvent (chloroform) as the dispersed phase and the aqueous phase of 10 w/w PVA and 0.1 M NaOH as a continuous phase to produce droplets in a glass capillary.^118^ At pH 8.5, by the diffusion of chloroform into the continuous phase and inversion of polymeric domains within the droplets, PLGA core forms in the p(BMA-*co*-DAMA-*co*-MMA) shell. Nile red (hydrophobic) and coumarin (hydrophilic) model drugs tend to accumulate in PLGA core and p(BMA-*co*-DAMA-*co*-MMA) shell, respectively. However, in pH 10, particles tend to form acorn-shaped Janus MPs (Fig. 7VI). Shell over multiple cores forms when the solvent is replaced with toluene over pH 8.5 to 10. Since the evaporation of toluene happens very fast (2.4-fold faster than chloroform), coalescence of PLGA domain stay separately during evaporation.

PLGA and poly(3-caprolactone) (PCL) MPs with patchy and Janus structures have been produced in droplet microfluidics in order to control drug release.^119,120^ A solution of PLGA and PCL in DMC or DCM produced organic droplets in a continuous aqueous solution of PVA (2 wt%). The strategy to achieve various morphologies was switching between two organic solvents. Polymer solution in DMC produces MPs with Janus particles (average size 24.42 μm) while in DCM, core–shell (average size 47 μm) structure with PLGA in the core and smooth surface.^119^ They investigated the effect of solvent concentration and the mass ratio of PLGA : PCL on the final MPs morphology and provided a diagram in order to easily choose the ratio for the desired final product. For example, core–shell MPs generated with a higher portion of PLGA (PLGA : PCL as 7 : 3 or 5 : 5) and anisotropic patchy particles harvested in the lower portions (PLGA : PCL as 3 : 7 or 2 : 8). At an equal mass ratio of polymers, Janus particle and patchy morphology produced with a change in the DCM : DMC volume ratio 1 : 5 and 2 : 1, respectively.^120^

In another strategy, Kang *et al.*^117^ produced PLGA Janus particles in which both parts are PLGA. For that purpose, they used two solvents to produce MPs; ethyl acetate (EA) and silicone oil are good and bad solvents for PLGA, respectively. The O/W emulsion in glass capillary which consisted of PLGA dissolved in two solvents as oil phase while continuous PVA aqueous phase as water flow. Faster evaporation of EA compared with removal of silicon oil, in the MPs formation stage produces Janus shape. Researchers claimed that the diameter of each part Janus PLGA MPs is predictable using theoretical and mathematical calculations.

## 5. Opportunities and challenges

PLGA DDSs production has started since two decades ago. It's obvious the method and produced NPs have undergone significant improvements since the first production of PLGA based NPs in microfluidics. PLGA based NPs are being synthesized with advanced features such as targeting ligands, lipid layers, stimuli-responsive, co-loaded with drugs and imaging agents. Various microfluidic chips (*e.g.* 2D, 3D, arc, and origami) have been used to investigate effective factors in the production process such as flow ratio, mixing time and microchannel size. Droplet-based microfluidics is being employed for the synthesis of complex MPs. This category of microfluidics has offered a powerful platform to control size, size distribution, rapid processing and uploading of drugs with varying hydrophobicity and properties. MPs are being fabricated with core–shell structure and even with multiple cores and/or shell. Apart from the encapsulation of incompatible drugs and/or unstable agents with varying physicochemical properties, the core–shell structure can make it possible to achieve sustained and even sequential release.

Although the technology provides a wide range of opportunities, it is not free from limitations and drawbacks that can hinder its application in large scales.

The first important issue that should be addressed by prospective researches is the fouling of PLGA NPs during the precipitation process and clogging the micro-size channels. As a matter of fact, the microfluidically production of PLGA DDS is considered as a continuous process which avoids batch-to-batch variation. This feature has been arguably accepted as one of the successful aspects of the technology. However, scientists are struggling with a huge number of failed chips in the lab due to the clogging of the microchannels. Not only fouling of NPs but also problems are being reported related to the wettability and hydrophobicity issues in MPs production. Despite all attempts done to solve the problems, results are not admissible and show that microfluidics hardware and choice needs to be selected according to every synthesis process.

Moreover, the contribution of various mixers like Tesla micromixers has been introduced and examined before and/or after focusing section of the synthesis process in continuous microfluidics. Two-stage microfluidically synthesis has been reported to control the core and shell more precisely in the production inorganic NPs.^121,122^ Such multi-stage could be advantageous in the production of PLGA-based DDSs with sophisticated features. Moreover, screening and preclinical test of drugs in living cells are crucial steps in drug discovery, direct purification and cell treatment on the microfluidic platform could be considered as additional steps toward the increased efficacy and speed.

Furthermore, manipulation of small volumes of liquids in micro-size has been mentioned as an advantage of these apparatuses; however, it is a double-edged sword and could be a disadvantage at the same time. Low volume of liquids can cause a low throughput of the fabrication process that cannot meet the industrial and large-scale production demand. Although there are few reports of high production rates suitable for preclinical and clinical demands, researchers need to search a solution for this very crucial issue. Future microfluidics should be able to produce a higher amount of drugs without sacrificing accuracy and efficiency.

## 6. Conclusion

PLGA based drug delivery systems are being produced with various fabrication methods and nowadays there are plenty of them approved by FDA and also many ongoing preclinical and clinical types of research to make their way to industry. For this purpose, researchers from all around the world look for new routes to produce DDS with more sophisticated features to promote the production and also delivery efficiency. In this context, we accented various type of microfluidic systems used for the production of PLGA based drug delivery, properties, and applications of PLGA NPs and MPs fabricated by this technology.

All together and in spite of drawbacks, inspiring abilities of the technique hold a great potential to bring and add exciting features to drug delivery systems *e.g.* controlled release, regulated surface and flexibility, stimuli-responsive and *etc.* knowledge gained from numerous examples of represented PLGA based drug delivery systems prepared in the microfluidics can help researchers to select proper reactants, microfluidics type and process to fabricate their goal drug delivery system.

## Conflicts of interest

The authors declare that there is no conflict of interest.

## Supplementary Material
